# What to Believe? Impact of Knowledge and Message Length on Neural Activity in Message Credibility Evaluation

**DOI:** 10.3389/fnhum.2021.659243

**Published:** 2021-09-17

**Authors:** Lukasz Kwasniewicz, Grzegorz M. Wojcik, Piotr Schneider, Andrzej Kawiak, Adam Wierzbicki

**Affiliations:** ^1^Chair of Neuroinformatics and Biomedical Engineering, Institute of Computer Science, Maria Curie-Sklodowska University in Lublin, Lublin, Poland; ^2^Polish-Japanese Academy of Information Technology, Warsaw, Poland

**Keywords:** EEG, credibility, source localization, LORETA, classifiers

## Abstract

Understanding how humans evaluate credibility is an important scientific question in the era of fake news. Message credibility is among crucial aspects of credibility evaluations. One of the most direct ways to understand message credibility is to use measurements of brain activity of humans performing credibility evaluations. Nevertheless, message credibility has never been investigated using such a method before. This article reports the results of an experiment during which we have measured brain activity during message credibility evaluation, using EEG. The experiment allowed for identification of brain areas that were active when participant made positive or negative message credibility evaluations. Based on experimental data, we modeled and predicted human message credibility evaluations using EEG brain activity measurements with F1 score exceeding 0.7.

## 1. Introduction

The World Wide Web has been designed for low barriers of entry, enabling fast, and cheap publication of content. At the same time, the prevalent business model of the web provides high incentives for producing Web content that impacts opinions and beliefs of Web users. These commercial incentives are caused by the popularity of Web-based marketing and advertising. However, Web content affects not just our shopping decisions, but also decisions regarding our health, or politics. In this technical and economic environment, the spread of fake news has become an increasingly significant social problem (Sharma et al., 2019). Fake news disseminate through social media, Web-based newspapers, blogs, and regular Web pages.

Although combating fake news has been the focus of policy and scientific research since 2016, to date, little is known about why people believe in fake news. While factors that contribute to belief in fake news have been studied by social psychology (Rutjens and Brandt, 2018; Forgas and Baumeister, 2019), these results have been obtained from the declarative studies. Simply asking Web users whether they believe fake news, or indirectly inferring this conclusion from their behavior, cannot reveal the real reasons for such a decision. The response to a question about the believability of fake news also cannot be a basis for a certain conclusion that fake news was indeed credible, because of possible biases in the response.

Surprisingly, almost no previous research has attempted to directly measure brain activity to study basic processes occurring in brain during credibility evaluation. Previous research using EEG or fMRI has been devoted to lie detection (Wang et al., 2016; Meijer and Verschuere, 2017). This approach is based on the investigation of the brain activity of the author, and not the receiver of the message.

The focus of our research is the brain activity during evaluation of message credibility. It is a fundamental aspect of credibility evaluation that focuses on the content, and not on the source of a message. In many online scenarios, Web users must evaluate the credibility of content without knowing the content's author or source. Our goal is to identify brain areas and periods of brain activity that are most active or most important in the process of textual message credibility evaluation. This process relies on the individual, subjective perception of a Web user and can therefore be studied experimentally using EEG.

This basic question leads us to a more applicable goal: creation of a method for EEG-based message credibility evaluation based only on the observed brain activity. In the future, we envisage the use of EEG for either testing the credibility of information in the form of fake news, or (to the contrary) correcting information designed to counteract fake news. Similarly to the use of EEG in online marketing (Deitz et al., 2016; Guixeres et al., 2017), researchers could evaluate in such a setting the credibility of information using a panel of information consumers.

The goal of this article is to address the following research questions:

- What brain areas are active while a receiver is evaluating message credibility?
- Does brain activity during credibility evaluation depend on message design?
- Can we model and predict human message credibility evaluations using EEG brain activity measurements?

One of the difficulties in addressing these questions lies in the fact that message credibility evaluation can be affected by two competing factors: message design and prior knowledge of message recipients. An experiment for studying message credibility must control prior knowledge of experiment participants about the message, as well as other factors that may influence message credibility evaluation. In this article, we describe an experiment that enables the study of message credibility evaluation without prior knowledge, and with perfect knowledge. In the former case, the message credibility evaluation of experiment participants can be influenced by irrelevant factors of message design. This situation reflects the reality of many Web users who encounter fake news on various subjects.

## 2. Related Work

### 2.1. Basic Concepts: Credibility and Truth

The concept of credibility is grounded in common sense and in used in scientific research. Modern research on credibility is active especially in the field of psychology, media science and informatics (Viviani and Pasi, 2017). In research, credibility is usually understood as a perceived quality of individuals. The earliest theoretical work on credibility from the 1950s is due to Hovland and Weiss (1951), who distinguished between *source, message, and media credibility*.

Out of these three, source credibility and message credibility are a good starting point for a top-down study of the complex concept of credibility. These two concepts are closely related to the dictionary definition of the term “credibility” (Oxford Advanced Learner's Dictionary): “the quality that somebody/something has that makes people believe or trust them.” A part of this definition focuses on a person (“somebody”) and is close to the concept of source credibility. Another part is about “something”—the message itself. That part defines message credibility that frequently needs to be evaluated on the Web without knowing the source of the message.

Information scientists have studied credibility evaluations aiming at designing systems that could evaluate Web content credibility automatically or support human experts in making credibility evaluations (Wawer et al., 2014; Liu et al., 2015; Kakol et al., 2017). However, human credibility evaluations are often subjective, biased or otherwise unreliable (Kakol et al., 2013; Rafalak et al., 2014), making it necessary to search for new methods of credibility evaluation, such as the EEG-based methods proposed in this article.

The concept of truth is even more complex than the concept of credibility. Without going into details (the reader is referred to Wierzbicki, 2018 for a detailed discussion), there exist several conflicting definitions of truth, such as scientific truth or post-structuralist truth. Truth may be also undecidable, or truth evaluation may be impossible in practice. However, the purpose of processes of disinformation verification and debunking is to discover information that is untrue and correct it by pointing out the truth. In this article, we shall assume a definition of truth as an objectively verifiable information that is the basis for disinformation checking and debunking.

Having said that, the relationship between credibility and truth is not simple. Non-expert web users may evaluate information that is not true as credible. We would expect that experts would evaluate only true information as credible. However, experts are human too, and can make mistakes. On the other hand, in many areas we have no choice but to rely on expert opinion, and to accept experts' credibility evaluations as truth.

### 2.2. Message Credibility

A search for the term “message credibility” on Google Scholar returns over 1,000 results (for an overview of recent publications, especially on the subject of Web content credibility, see Viviani and Pasi, 2017; Wierzbicki, 2018). Media science researchers have attempted to create scale for declarative measurements of message credibility (Appelman and Sundar, 2016). Message credibility has been investigated in the area of healthcare (Borah and Xiao, 2018).

Message credibility has been defined as a scientific concept by Hovland and Weiss (1951) as the aspect of credibility that depends only on the communicated message, instead of the message's source or communication medium. On the Web, the message is a webpage that includes an article (or a shorter text in case of social media). Message credibility depends on the textual content, on images or videos (as well as advertisements) embedded in the webpage, and on webpage design or style.

What follows is that message credibility is affected by many factors (features of the message). Even if we consider only the textual content, message credibility can be affected by semantic or pragmatic aspects of the message (its meaning and style, persuasiveness, sentiment etc.) This complexity is especially important because message credibility is usually evaluated rapidly on the Web.

Tseng and Fogg (1999) introduced the two concepts of “surface credibility” and “earned credibility”. Surface credibility is based on a fast and superficial examination of the message (similar to System I reasoning, as introduced by Kahneman, 2011). Earned credibility, on the other hand, is the result of a more deliberate and time-consuming evaluation of the message, like System II reasoning. Research (Wierzbicki, 2018) has established that most users evaluate webpage credibility quickly, usually requiring several minutes (3 min are enough for most Web page credibility evaluations). Earned credibility evaluation requires much more time and usually involves a debunking or verification process. These observations are relevant for our experiment design. In this article, we focus on surface credibility evaluations based on the contents of the message. In order to begin understanding brain activity during message credibility evaluation, we shall design messages that differ by a single aspect that can be evaluated quickly.

Message credibility evaluation of Web content is often difficult for ordinary Web users. This problem has led to numerous attempts of designing automated or semi-automated IT systems that support Web content credibility evaluation (Viviani and Pasi, 2017; Wierzbicki, 2018; Sharma et al., 2019). In this article, we focus on how humans make message credibility evaluations without computer support.

### 2.3. Experimental fMRI and EEG Findings

Research on brain signaling in decision-making focuses on neuroimaging (fMRI) and the activity of specific parts of the brain in the situation when participants solve various tasks.

Many of these studies concern confidence in the person, as in facial plausibility studies in which Amygdala activity has been demonstrated (Rule et al., 2013), also as Precuneus-the medial part of Brodmann Area (BA) 7, Inferior Frontal Gyrus—BA44, BA45, BA47, Medial Prefrontal Cortex—BA12, BA25, BA32, BA33, BA24 (Filkowski et al., 2016). The dynamic role of the Paracingulate Cortex(BA9/32) and Septal Area in supporting conditional and unconditional trust strategies in Trust Games was investigated (Krueger et al., 2007). Also difference in neural activation (BA7, BA8, BA40) between prosocials and proselfs people during decision making and interaction effect between dispositional trust and social value orientation (BA9, BA31, BA39) was shown in Emonds et al. (2014).

Few of these studies are concerned with trusting the message itself. In the Processing of Online Trust Signals study, online shopping activity of Rolandic Operculum was reported with the most trustworthy signal (BA 44 is part of it), calcarine—is where the Primary Visual Cortex is concentrated (BA17), Angular Gyrus (BA39) and Superior Motor Area, pre-SMA (BA 8) (Casado-Aranda et al., 2019).

Anterior cingulate cortex (BA24, 32, 33) activity was observed, but only within high effort condition such as the quickest possible pressing of the button task (Mulert et al., 2008) in the decision-making study on the pitch of the tone.

Heekeren et. al. suggest that Posterior Superior Temporal Sulcus and Ventromedial Prefrontal Cortex (BA10) are involved in decision-making regarding scenarios devoid of violence and direct bodily harm (Heekeren et al., 2003).

In terms of EEG, the N1 and P300 signals combined with fMRI data in value-based decision-making were examined (Mulert et al., 2008; Larsen and O'Doherty, 2014). In this study, decision making involved activation of Dorsomedial Prefrontal Cortex (BA8, BA9, BA10, BA24, and BA32) and Ventromedial Prefrontal Cortex (BA10).

In a study by Douglas et al. (2013) with the help of ICA, the authors managed to create satisfactory models using EEG signal (power envelopes derived from spectral bands as features) in classification of belief/disbelief decision-making.

Areas such as BA.08, BA.09 have been proposed to participate in a general mechanism for perceptual decision-making in the human brain (Heekeren et al., 2004).

## 3. Experiment Design

### 3.1. Motivation for Experiment Design

As a practical reference situation that motivates our experiment design, consider a receiver of a message on social media. The message could be true, or it could be disinformation. The receiver evaluates the surface credibility of the message. This means that she will quickly (within a matter of minutes or even seconds) decide whether the message is credible or not, and act accordingly (by forwarding, retweeting, or liking the message). This kind of situation is so common that experts on media literacy have coined the slogan: “think before you like” (Harrison, 2017). Fast, superficial credibility evaluation is not only common, but can lead to innumerable social harm, especially in the context of health-related Web content.

For the sake of our reasoning, consider that the message contains information related to health or medicine (about 60% of Americans and Europeans go online looking for health information; Viviani and Pasi, 2017). It could be a simple statement like “Low doses of aspirin can be safely consumed in the second trimester of pregnancy” to increasingly complex statements, for example: “Coenzyme Q10 supplements may help prevent statin side effects in some people, though more studies are needed to determine any benefits of taking it.” For the record, the first statement is generally true, especially if there is risk of miscarriage. The second statement is mostly false and contains a hedging part to make it more credible; it is designed to sell coenzyme Q10 supplements, but can also discourage the use of statins by people who need them because of high cholesterol and arteriosclerosis.

Please note that the first factor that impacts a receiver's credibility evaluation of such a statement is the receiver's knowledge and experience. Controlling this factor is therefore a crucial element of our experiment design. However, it is hard to evaluate degrees of knowledge and control their impact on credibility evaluation. For this reason, we designed two experimental conditions: full knowledge and lack of knowledge. Lack of knowledge is particularly characteristic of online situations, such as when a non-expert social media user is evaluating a message concerning health or medicine. The full knowledge scenario is more applicable to credibility evaluations by experts.

If the message is just a simple textual statement, the second factor that can influence credibility evaluation is the message's persuasiveness. This factor is even harder to understand and control. Contemporary research on Natural Language Processing for detecting persuasive disinformation uses complex language models, and achieve accuracy of 70–80% (Wawer et al., 2014). For experiment design, this is insufficient. Moreover, persuasive disinformation can influence many different cognitive biases or heuristics—they can appeal to positive or negative emotions, use hyperbolization, forgery, selective presentation of information, and many other techniques. In a single experiment, it would be impossible to consider all of them, and focusing on a single, yet complex technique is contrary to our research goal of achieving a generalizable understanding of how the brain processes message credibility evaluation.

For this reason, in our experiment we decided to use a simple factor that differentiates between messages—the message's complexity. Research has found that this factor has a significant impact on message persuasiveness (Wawer et al., 2014; Kerz et al., 2021). We use short messages that consist of one or a few words, and long messages that give a detailed description of the translated kanji characters. Results of our pilot experiment (Kwaśniewicz et al., 2020) (and of the main experiment itself—see section 4.1) indicate that receivers are influenced by message complexity and tend to positively evaluate message credibility of long messages more frequently than of short messages. Message credibility evaluation without knowledge, affected only by message length, is not random, as evidenced by statistical tests.

### 3.2. Controlling Participant Knowledge

The goal of the experiment was to observe electrical activity and the most active areas of the participant's brain cortex during tasks involving message credibility evaluation, as well as the influence of the message design and content on this process. In order to ensure that participants could only rely on message design during the experiment, it was designed so that the participants would not be familiar with the topic of the messages. Selected topic of the messages concerned the meaning of Japanese kanji signs.

The experiment was designed to create a situation in which the participants assess truthfulness or falsehood with practically no prior knowledge of the message subject. Knowledge of participants about the correct meaning of Kanji signs used in experiment was controlled. The initial condition ensured that participants had no knowledge of Kanji signs^1^. However, the participants were taught the meaning of three Kanji signs. When they were shown a Kanji sign unfamiliar to them, experimental setting resembled the case when a person who has no knowledge of the subject receives fake news. In order to study the effect of knowledge, we also showed them the previously taught Kanji signs.

Note that instead of Kanji signs, we could have used other images (for example, USG scans of different types of tissue). Our choice of Kanji signs was motivated by the fact that this type of image has been extensively studied using EEG (Sakurai et al., 2000; Ardila et al., 2015; Higashino and Wakamiya, 2021), and we knew from literature what brain activity to expect from participants who examined Kanji signs.

### 3.3. Participants and Ethical Commission's Permission

The participants to the experiment were right-handed male students without any knowledge of Japanese. A total of 107 participants took part in the experiment. EEG signal from 105 participants was collected. The experiment was carried upon the permission of the University's Bioethical Commission (MCSU Bioethical Commission permission 13.06.2019).

### 3.4. Message Credibility Evaluation Task

In the first part of the experiment, participants were requested to learn three Japanese Kanji signs by heart: eye,mouth and mountain. These characters were chosen because of their simplicity and a lack of similarity with other characters, making it simple for participants to correctly identify them. In fact, during the entire experiment, no participant made even a single mistake during the identification of these characters.

In the next step, participants saw a single message on the screen that contained a translation of a Kanji sign into their native language. They had to answer whether they considered the translation of the Kanji sign to be true or not. Participants had to answer in a maximum of 3.5 s from the appearance of the translation.

**Figure 1:**
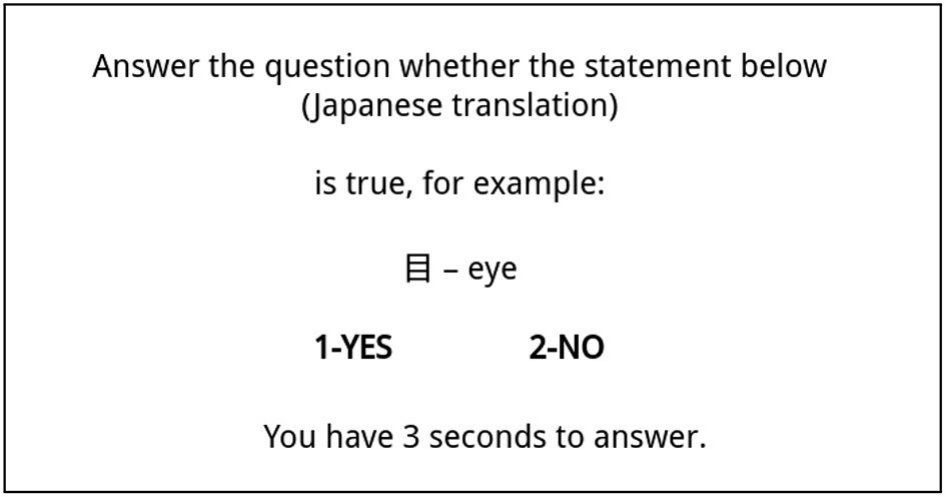
Typical screen shown to participant during the experiment. In this case participant was to decide whether this Kanji sign means Eye, having perfect knowledge about it.

The decision task was formulated in the form of a question: “Is this Japanese translation true.” The participants could answer the question by selecting “Yes” or “No.” The choice of the answer “yes” was equivalent to a positive evaluation of message credibility. An example screen is shown on Figure 1. There were 260 such questions in total.

### 3.5. Controlling the Language Complexity of the Message

**Figure 2:**
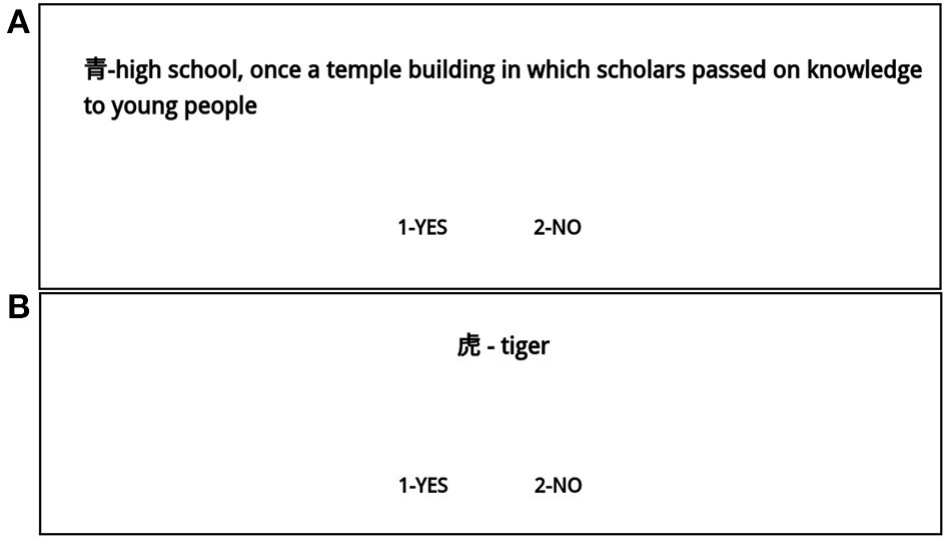
Examples of screens with a long **(A)** and short **(B)** notes used in experiment for translation of Kanji sign.

The proposed translations shown to the participants differed in the length of the explanation of the Kanji sign. The meaning of 160 translations was explained in a single word, while the meaning of the remaining 80 translations was explained in a full sentence in the participants' native language. Longer explanations included were designed to give additional detail or to logically explain the relationship between the shape and meaning of the Kanji sign. For examples, see Figure 2. For brevity, we refer to the single-word Kanji sign translations as “short note,” and to the longer translations as “long note.”

### 3.6. Experimental Cases and Data

There were 240 screens shown to each participant during the experiments.

The first group of 80 screens contained one of the three Kanji signs that the participants have learned before, described by a short note. Therefore, the participants had perfect knowledge of them. This first group of 80 screens consisted of 40 screens with correct translations and 40 screens with incorrect translations.

In the second group, there were 80 screens with Kanji signs that were completely unknown to the participants, described by a short note. In the third group, there were 80 screens also with completely unknown Kanji signs, described by a long note.

It should be noted that there was no group of screens with perfectly-known Kanji signs described by a long note. The reason for this is that for the perfect knowledge cases, we did not wish to confuse users with other factors that could have an impact on their message credibility evaluations.

Such a setup allowed us to register electroencephalographic activity in the following six cases of choice:

1. TT: true translation of a known Kanji sign was evaluated as credible
2. FF: false translation of a known Kanji sign was evaluated as not credible
3. ST: short translation of an unknown Kanji sign was evaluated as credible
4. SF: short translation of an unknown Kanji sign was evaluated as not credible
5. LT: long translation of an unknown Kanji sign was evaluated as credible
6. LF: long translation of an unknown Kanji sign was evaluated as not credible

In this experiment, participants were not mistaken in questions about known Kanji signs, and no signal has been registered for the following hypothetical cases:

- true translation of a known Kanji sign was evaluated as not credible (TF)
- false translation of a known Kanji sign was evaluated as credible (FT)

This means that in our experiment, we can consider the cases when participants knew the Kanji signs as a model of message credibility evaluation with perfect knowledge.

### 3.7. Hypotheses

We formulated the following hypotheses:

1. Length of the note has a significant positive influence on the participant's decision about message credibility.
2. Decision of participants about message credibility in all three cases (short note with or without previous knowledge, and long note without previous knowledge) can be predicted based on measurements of mean electric charges in participant's brains.
3. Length of the note has a significant influence on brain activity during making decisions process concerning message credibility, and there are some significant differences in the models predicting decisions of participants who had seen long note compared to participants who had seen short notes.
4. Previously learned knowledge of the shown Kanji sign had a significant influence on brain activity during making decisions process concerning message credibility and there are some significant differences in the models predicting decisions of participants who had previously knowledge as compared to participants who did not have this knowledge.
5. There are some significant differences in the models predicting decisions of participants who frequently choose long note as compared to models of other participants.
6. There is a satisfactory model to predict the participants' decision based on the left Brodmann Areas 08,09 described in the literature as related to the decision-making process.

Hypothesis 1 is not directly related to participants' brain activities. It is rather a test of our experiment's internal validity. Positive validation of hypothesis 1 would confirm that there is a relationship between one of the main independent variables of our experiment and the participant's decision. Such a relationship would partially confirm the internal validity of our experiment.

Hypothesis 2 can be validated by constructing classifiers that predict the (binary) decision of participants with sufficiently high accuracy. However, the validation of hypothesis 2 requires training of three classifiers: first based on the set of participants who evaluated message credibility based on their knowledge (this classifier would have two classes: TT and FF), second classifier based on message credibility evaluations made without knowledge (classes: ST and SF), and third one based on the set of participants who made decision under the impact of message design (LT and LF).

Hypothesis 3 is related to the first and second research question. It is focused on differences in the brain processes during credibility evaluation processes under the impact of message design (length). To validate this hypothesis, we need to compare two models based on situation when short note is evaluated (classes ST and SF) and when long note is evaluated (classes LT and LF). To enable this comparison, the classifiers trained for validating hypothesis 2 should be interpretable (based on logistic regression, decision trees or a similar method).

Hypothesis 4 concerns the effect of other main independent variable: previous learned knowledge of the Kanji sign. The experiment design allowed us to control this variable: our participants had perfect knowledge or no knowledge. We can therefore study the effect of knowledge on message credibility evaluation process. A validation of this hypothesis requires a comparison between two different classification models: one for classes TT and FF, and the other for ST and SF.

Similarly, the validation of hypothesis 5 requires training of two classifiers, one based on the set of participants who tend to evaluate long messages as credible, and another one based on the remaining set of participants. The comparison of these two classifiers is only possible if the two of them are explainable, which excludes the use of black-box classifiers such as neural networks.

Hypotheses 6 can be validated by constructing classifiers that will use signal from left Brodmann Areas 08,09 and predict the (binary) decision of participants with sufficiently high accuracy.

### 3.8. EEG Measurements

Our empirical experiments involved top EEG devices. We were equipped with a dense array amplifier recording the cortical activity with up to 500 Hz frequency through 256 channels HydroCel GSN 130 Geodesic Sensor Nets provided by EGI^2^. In addition, in the EEG Laboratory the Geodesic Photogrammetry System (GPS) was used.

Estimating ERP for each of the 256 electrodes is not necessary for ERP observation, as in general standards there are just a few electrodes (in our case 26) playing an important role in cognitive tasks^3^. However, for the sLORETA source localization analyses (used for verification of the next hypotheses) the ERP for all 256 electrodes had to be in fact calculated on the fly.

Having the ERP signal estimated for each electrode out of 256, it was possible to calculate the mean electric charge (MEC) flowing through the BA situated under these electrodes on the brain cortex in cognitive processing time interval (CPTI) as described in Wojcik et al. (2018) and Kawiak et al. (2020). Moreover, it was also possible to conduct the full source localization analysis of the signal originating from all 256 electrodes using sLORETA algorithm (GeoSourse parameters set as follow: Dipole Set: 2 mm Atlas Man, Dense: 2,447 dipoles Source Montages: BAs). Mean electric current flowing through each BA and varying in time was given as an output. Having those values calculated, it was possible to integrate that current in time and then get the MEC. The mean electric charge calculated for each electrode using source localization techniques could, as we intended, indicate the hyperactivity of some BAs that are not necessary precisely situated under the cognitive electrodes. For all calculations of MEC, the CPTI was divided into 5 ms time intervals. The procedure of calculating MEC has been described detail in Wojcik et al. (2018).

## 4. Experiment Results

### 4.1. Impact of Note Length on Message Credibility Evaluations Without Prior Knowledge

As described in section 3.3, we have collected sufficient data to measure ERP and execute source localization from 105 male, right-handed participants. Signal was collected from 105 people in cases TT∪FF, 104 in cases ST∪SF, and 95 in cases LT∪LF. One person finished experiment after the first part (TT∪FF), while nine others did not answer the question about long notes (LT∪LF).

The impact of note length on message credibility evaluations can be established by comparing four experimental cases: LT, LF, ST, and SF. In particular, hypothesis 1 states that we expect that note length has a significant positive influence on the participants' decision about message credibility. Thus, we expect that


(1) $$\begin{array}{l} \frac{\left|LT\right|}{\left|LT|+|LF\right|}>\frac{\left|ST\right|}{\left|ST|+|SF\right|} \end{array}$$

To check whether this condition is satisfied, we only needed to count the number of experimental results in the four cases. There were 4,079 results when participants evaluated long translation of an unknown Kanji sign as true (LT case) and 3,526 results when participants evaluated long translation of an unknown Kanji sign as false (LF case). This gives a proportion of


(2) $$\begin{array}{l} \frac{\left|LT\right|}{\left|LT|+|LF\right|}=53.6\% \end{array}$$

positive message credibility evaluations of long notes. Correspondingly, there were 3,455 experiment results when participants evaluated short translation of an unknown Kanji sign as true (ST case) and 4,977 experiment results when participants evaluated short translation of an unknown Kanji sign as false (SF case). The resulting proportion of positive message credibility evaluations of short notes is


(3) $$\begin{array}{l} \frac{\left|ST\right|}{\left|ST|+|SF\right|}=40.9\% \end{array}$$

which is a significantly lower number than for long notes. This observation positively verifies Hypothesis 1.

We also tested whether or not the message credibility evaluation was random in the cases without knowledge. Participants could evaluate the long or short note as credible. If the choice would be random, the choices of the long or short note should form a binomial distribution with probability 0.5. We used the binomial test and calculated the p-value, which was <0.000001 (we observed 3,455 choices of the short note out of 7,534 message credibility evaluations). Therefore, we concluded that we could reject the possibility that the choices of short or long notes in the experiment cases without knowledge were binomially random.

### 4.2. Method for Selecting Independent Model Variables

In order to verify hypotheses 2, 3, 4, 5, 6 we needed to build machine learning models of message credibility evaluations in our experiment.

First step toward the creation of machine classification models consisted in the selection of independent variables. While it would be possible to use the MEC from all Brodman Areas in all time intervals to define independent variables, such a model would most likely be overfitted, and would also have a minimal capacity for interpretation. Therefore, we followed a special method for selecting a smaller subset of independent variables of the model based on the MEC from various Brodman Areas.

**Figure 3:**
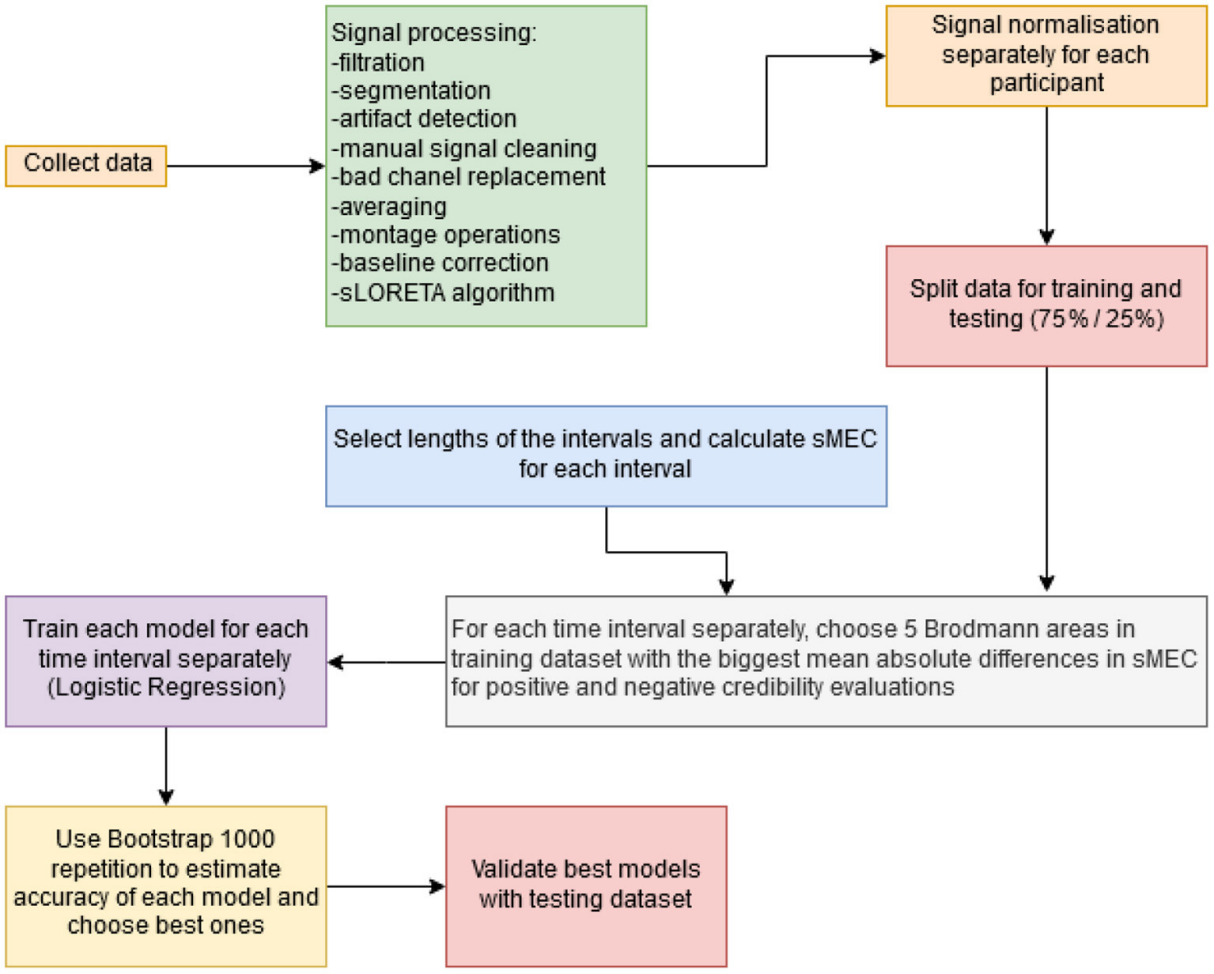
Data processing steps in analysis of experimental results.

All steps of the method for selecting independent variables are shown in Figure 3. First step is state-of-the-art EEG signal processing and the use of the sLORETA algorithm for source location. In contrast to our earlier research (Wojcik et al., 2018; Kwaśniewicz et al., 2020) that used the mean electric charge (MEC) flowing through each BA, in our new approach, the MEC signal was normalized to the range from 0 to 1 for each BA of every participant. Brain cortex is covered with the mantle of meninges, bones of the skull, skin and hair which results in a different SNR. Therefore, the electrophysiological activity of particular participants may be different in its measured power. To avoid the impact of such individual differences, we propose to normalize the MEC values. The normalized (scaled) MEC will be referred to as sMEC.

In the next step, the dataset was divided into training and validating datasets. There were 78 participants in the training dataset and 27 participants in the validating one for TT and FF classification, 78 (training) and 26 (validating) for ST and SF classification, and 74 (training) and 21 (validating) for LT and LF classification. This split was performed to avoid any possible data leakage during the testing of the models. The remaining steps of independent variable selection were done on the training dataset. This means that the model was designed and trained using only training data, and finally tested on a separate testing dataset.

An important step within the method was the selection of time intervals for MEC calculation. Time intervals were selected taking into account all possible lengths and offsets from stimulus, with a resolution of 5 ms. This means that a considered time interval could be [0, 25 ms] from stimulus, or [0, 990 ms] (the entire duration of the experiment)—as well as all possible combinations of the beginning and ending time, with a resolution of 5 ms. The choice of 5 ms intervals was then found to be optimal for classifier efficiency in such a temporal resolution turned out to be the best for achieving satisfactory classification results. Choosing higher time resolution would result in averaging the MEC changes and the smaller resolution would be too tiny for observation statistically significant differences in activity.

It was assumed that the difference in decision making would be reflected in the signal of brain activity. It was not known how long it took the participants to make a decision or when exactly it was made before the response pad was clicked. The 5 ms interval turned out to be insufficient to follow the decision. The comparison of sMEC from different time intervals made it possible to select those intervals with the largest mean value differences for the selected classes. The selection of the time intervals for which the sMEC was calculated was made only from the training data set, preventing data leakage. The validation of the classifiers on the testing set with good accuracy results, positively verified the correctness of selecting such explanatory variables.

For every selected time interval, we calculated sMEC for all Brodmann Areas, for all responses. Next, we chose up to 5 Brodmann Areas that had the largest mean absolute difference in sMEC for positive and negative credibility evaluations (for the two classified cases). We limited the maximum number of explanatory variables to 5, because in our previous studies, logistic regression models using five Brodmann's Areas had been as accurate as those using more areas (Kwaśniewicz et al., 2020).

For 5 Brodmann Areas, there could be 39,175,752 models (all combinations without repetitions of 5 Brodmann Areas out of 88) for a single time period. The number of time intervals to be checked depends on the selected length, e.g., for 25 ms we had 194 intervals to be checked for timeline 0–900 ms and the smallest possible interval as 5 ms. From the above selected lengths, we obtained 2,261 time intervals, and 391,758,752 combinations. This means that there were 88,576,375,272 models to check. Since this number was too large, we limited the number of combinations of Brodmann Areas by only considering the 5 Brodmann Areas with the largest mean absolute difference in sMEC for a given time interval between the two classes as in the following equation:


(4) $$N=I\sum_{i=1}^{Q}\left(\begin{array}{l} Q\\ i \end{array}\right)$$

where Q = 5 and I = 2,261.

This limiting approach left us with possible 70,091 models. Out of these, we selected the model with the best accuracy that was estimated with the use of the bootstrap validation method (1,000 repetitions) on the training data. Finally, the accuracy of the chosen model was evaluated on the validating dataset. It should be note that the models could have up to five explanatory variables that are sMEC values from the chosen Brodmann Areas in the selected time interval.

### 4.3. Machine Classification Models of Message Credibility Evaluations

The logistic regression classifier was implemented in R language using the stats v3.6.3 library.

Results are shown in Table 1. The best models achieved an accuracy of at least 0.7 on the training and validating datasets, which confirms hypothesis 2. Overall, the best results were achieved for the classification of the ST and SF cases (short notes without knowledge). However, the best models for the different classification problems differ significantly with respect to the time interval and the Brodmann Areas selected to determine the independent model variables.

**Table 1:** Best models' scores for each case used for verification of hypothesis 2.

**Message case**	**Intervals (ms)**	**Brodmann areas**	**Bootstrap accuracy**	**Validation accuracy**	**Validation precision**	**Validation recall**	**Validation f1**
Short note	105–330	R.BA.46, R.BA.0402	0.70	0.79	0.78	0.81	0.79
UNKNOWN		R.BA.36,L.BA.33					
(ST and SF)		L.BA.					
Short note	330–530	R.BA.39, L.BA.370.71	0.71	0.70	0.69	0.74	0.71
KNOWN		L.BA.25,R.BA.31					
(TT and FF)							
Long note	830–855	L.BA.31, L.BA.44	0.70	0.74	0.67	0.78	0.72
UNKNOWN		R.Hippocampus					
(LT and LF)		L.BA.09, R.BA.08					

In particular, different time intervals and different Brodmann Areas were used in the best model for long messages, and best model for the short ones, which supports the Hypothesis 3. Similarly, Brodmann Areas and time intervals differ for the best models for known and unknown messages, which confirms the Hypothesis 4.

**Figure 4:**
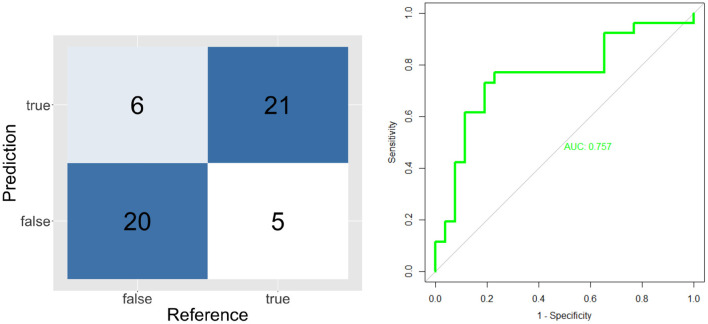
Confusion matrix and ROC curve for short unknown message's model (ST and SF).

**Figure 5:**
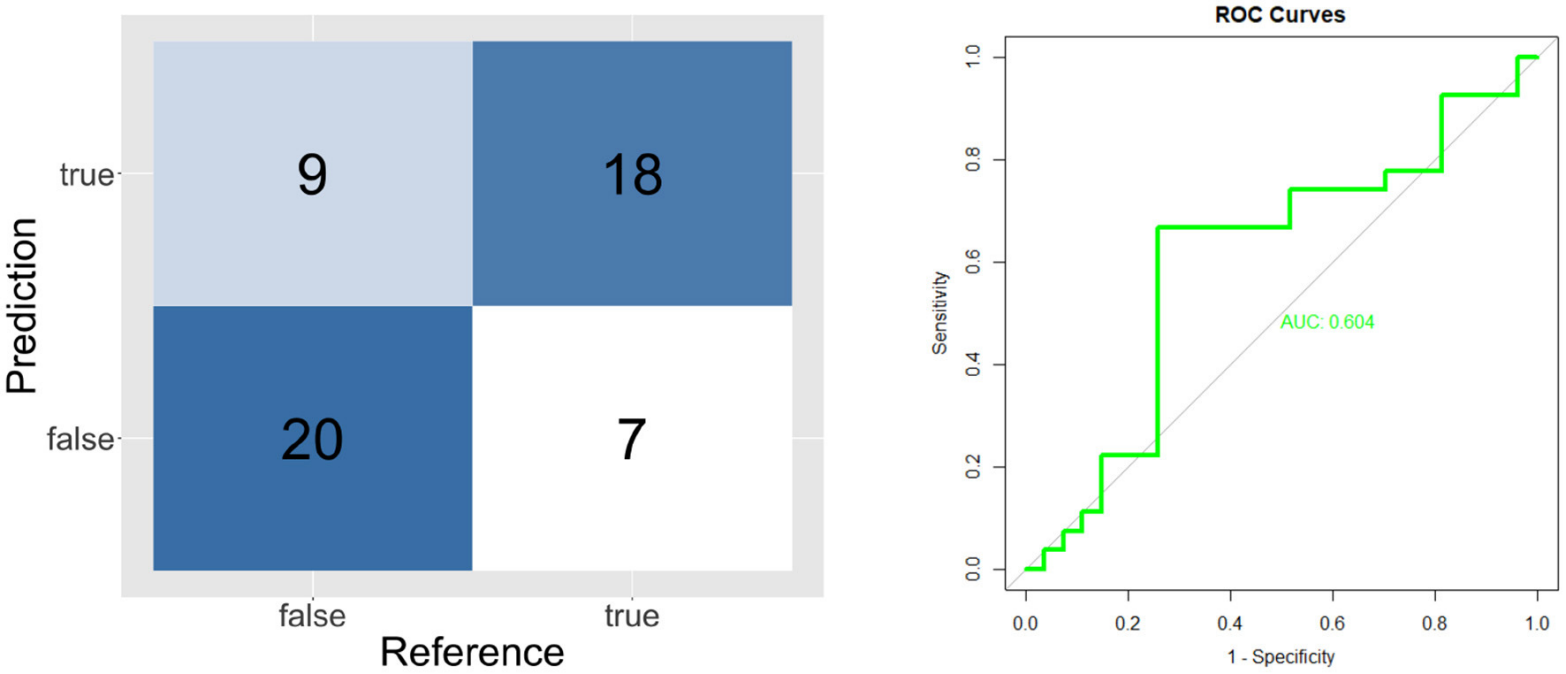
Confusion matrix and ROC curve for short known message's model (TT and FF).

**Figure 6:**
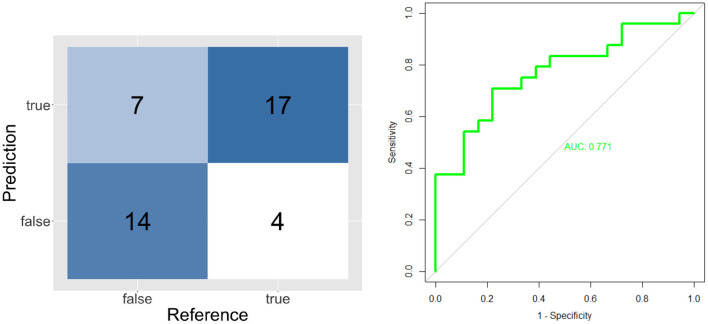
Confusion matrix and ROC curve for long unknown message's model (LT and LF).

The validation results of individual classifiers are shown in the form of confusion matrices, ROC curves and Area Under the Curve on Figures 4–6.

**Figure 7:**
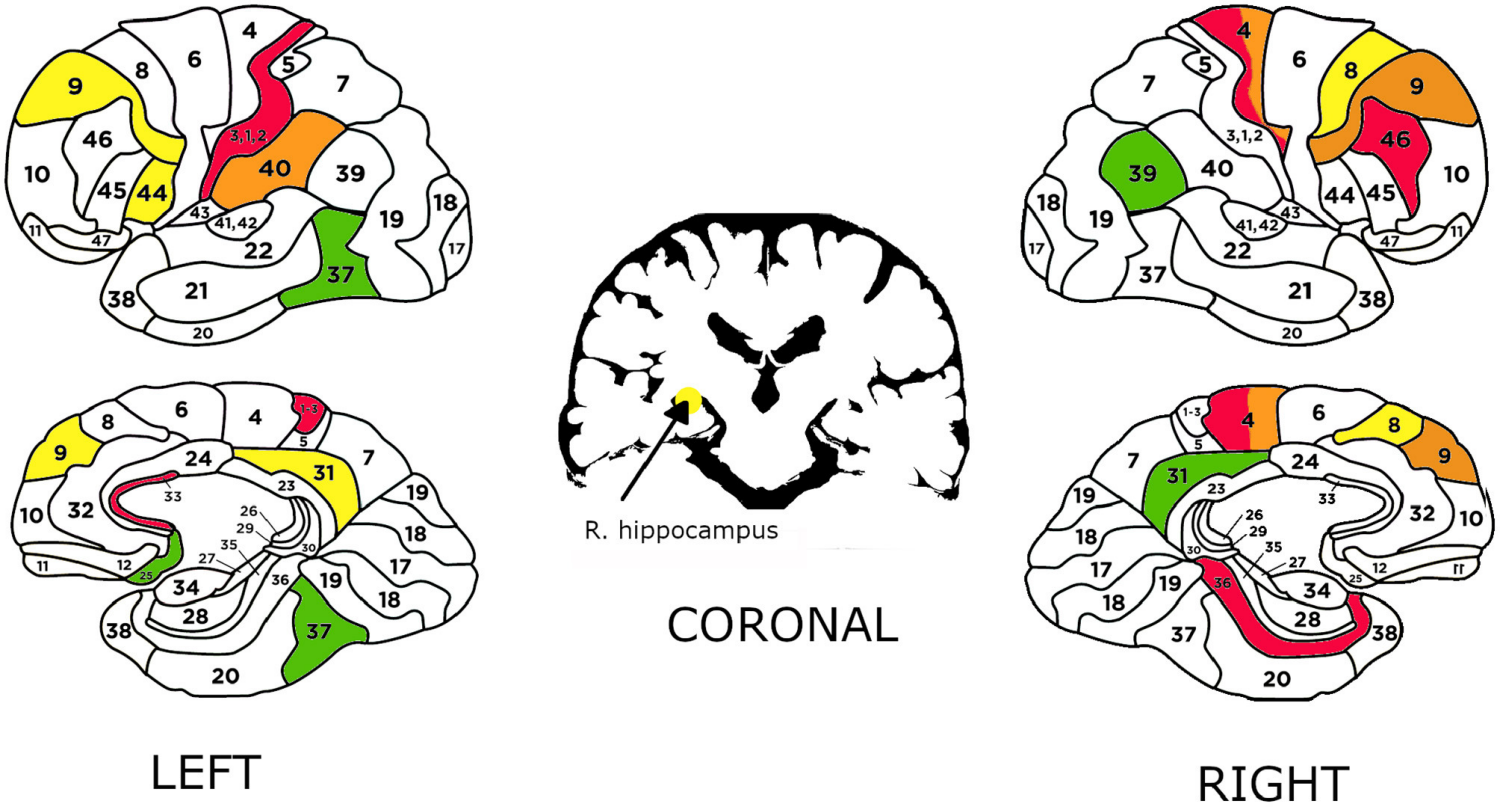
Brodmann areas (left and right view) and hippocampus (coronal view) used in the best classifiers. The colors: green, yellow, orange, and red correspond to the classifiers in the following order: TT and FF, LT and LF (entire population), LT and LF (long note prefered), ST and SF.

All areas used in the classifiers are shown in Figure 7. In the case when participants evaluated message credibility without prior knowledge, the best model used a signal in the range of 105-330 ms. The model used Brodmann Areas such as:

- BA.46 which is the area in which Fleck et al. (2006) observed increased activity in decision making under uncertainty
- BA.36—activity of this area was observed when listening to a foreign language (Perani et al., 1996)
- BA.02, BA.04—motor sensor areas, changes in these areas have been observed associated with error processing in the context of visual feedback (Wilson et al., 2019)
- Another area is BA.33—a part of Anterior cingulate cortex. Basic theory states that the Anterior cingulate cortex is involved in error detection (Bush et al., 2000). Evidence for this conclusion has been derived from studies involving a Stroop task (Posner and DiGirolamo, 1998).

When the participants had knowledge about the meaning of Japanese characters, the model used the signal from a later time interval: 330–530 ms and Brodmann Areas such as:

- BA37—a common node of two distinct networks-visual recognition (perception) and semantic language functions (Ardila et al., 2015)
- BA.39—involved in language reception and understanding (Ardila et al., 2016)
- BA.31—studied in case of decision-making in perceptual decisions (Heekeren et al., 2004)
- BA.25—considered a governor for a vast network involving areas like hypothalamus, brain stem, amygdala, and hippocampus (Ressler and Mayberg, 2007)
- BA.44—involved in processing of different types of linguistic information (Heim et al., 2009).

Also, areas like BA.08, BA.09, BA.31, Hippocampus have been linked to decision making (Maddock et al., 2003; Heekeren et al., 2004; Deppe et al., 2005; Volz et al., 2005; O'Neil et al., 2015).

Areas such as BA.08, BA.09 have been proposed to participate in a general mechanism for perceptual decision-making in the human brain (Heekeren et al., 2004).

#### 4.3.1. Cognitive Bias for Long Notes

In our experiment, message credibility evaluation could be affected by an irrelevant factor: the length of the message. Hypothesis 5 concerns the existence of differences in the models obtained for participants who frequently evaluated long note as credible, as compared to other participants. To verify the hypothesis, we needed to identify participants who evaluated long notes as credible more frequently than short notes. In order to do this, we calculated for each participant how many times that participant evaluated long notes as credible (LC) and how many times the same participant evaluated short notes as credible in the absence of prior knowledge (SC). Next, for each participant we calculated the ratio *LC*/*SC*. This ratio indicates how much more frequently the participant evaluated long notes as credible, as compared to short notes.

Forty-seven participants had a *LC*/*SC* ratio above the mean value of all participants. We have followed the same procedures as described above to build a model that classified the cases LT and LF for participants in this group.

The best model for the classification of *LT* and *LF* for participants who prefer long notes is different from the model for all participants, as shown in Table 2 and its best coefficients in Table 3. The model for participants who prefer long notes does not use the areas L.BA.31, L.BA.44, R.Hippocampus, R.BA.08. Both models also use other language networks.

**Table 2:** Models for classification of message credibility evaluations of long notes without prior knowledge (LT and LF cases) for participants who preferred long notes and for the entire population.

**Case and group**	**Intervals (ms)**	**Brodmann areas**	**Bootstrap accuracy**	**Validation accuracy**	**Val precision.**	**Val recall.**	**Val f1.**
LT and LF long note preferred	800–875	L.BA.40, R.BA.04 R.BA.09	0.71	0.73	0.7	0.7	0.7
LT and LF entire population	830–855	L.BA.31, L.BA.44 R.Hippocampus L.BA.09, R.BA.08	0.70	0.74	0.67	0.78	0.72

*Boostrap and validation accuracies are presented in columns 4 and 5*.

**Table 3:** Coefficients in logistic regression for best models.

**ST and SF**		**LT and LF**		**TT and FF**	
**Variables**	**Coefficients**	**Variables**	**Coefficients**	**Variables**	**Coefficients**
Intercept	−3.23286	Intercept	0.4133	Intercept	−2.82279
R.BA.46	0.08884	L.BA.31	0.4360	R.BA.39	0.09693
R.BA.04	0.05689	L.BA.44	−0.3214	L.BA.37	0.03943
R.BA.36	0.06321	R.Hippocampus	−0.2790	L.BA.25	0.06532
L.BA.33	0.02682	L.BA.09	0.4657	R.BA.31	−0.02018
L.BA.02	0.07420	R.BA.08	−0.4305		

BA40 is part of a language reception/understanding system and it is involved in language associations (associating words with other information) (Ardila et al., 2016). BA44—language production system. BA 44 supports modality-independent lexical decision making (Heim et al., 2007). Hippocampus is associated with declarative memory, including the memory of facts (Squire, 1992). Overall findings showed that Hippocampus may be part of a larger cortical and subcortical network seen to be important in decision making in uncertain conditions (O'Neil et al., 2015), some studies show that activity in regions BA 9, BA 31, increases with increasing trust as well (Emonds et al., 2014). Research also indicates activation of BA 8 reflects that we are uncertain (Volz et al., 2005).

These results may indicate smaller uncertainty and distrust associated with a negative message credibility evaluation of a long note by participants who prefer long notes. Further work should consist in creating a model using the EEG signal to classify the confidence level of the participants who evaluate long notes as credible.

Changes in areas BA.9, BA.25, BA.31, BA.44 (Altshuler et al., 2008; Klempan et al., 2009; Goodman et al., 2011; Alexander et al., 2019), may also be related to mental illnesses such as depression. Kim et al. (2012) indicates that low interpersonal trust appears to be an independent risk factor for new-onset and long-term depression.

### 4.4. Discussion and Limitations

For each case—short known message, short unknown message, and long unknown message, we received different best models that used sMEC from differing time intervals and Brodmann Areas.

The Brodmann Areas, used by our models, confirm previous studies in which the activity of these areas was observed in decision-making processes under uncertainty, or it was related to language processing.

Our method of model design relies on choosing time intervals that had the largest differences in brain activity for the classified cases. Optimal time intervals chosen for classifying pairs of cases: ST and SF, TT and TF, LT and LF were: 105–330, 330–530, and 830–855 ms, respectively. Significant differences in these time intervals correspond to the nature of the task for each pair of cases. First pair, ST and SF, is message credibility evaluation of short notes without prior knowledge. In this case, models used the earliest time interval, which indicates that brain activity crucial for making decisions occurred quite quickly after the stimulus. On the other hand, second pair of cases, TT and TF, involved message credibility evaluation with prior knowledge. In this case participants needed more time, likely for comparing messages to memorized facts and making decisions based on comparison results. Last pair of cases based on message credibility evaluation of long notes without prior knowledge. Late time interval was most likely caused by the subjects having to read a longer text.

Using the same time intervals as in our models for the same cases, we created models consisting of the signal from the left BA08 and BA09 given as areas involved in the decision making process. The results we obtained were less effective than the results of the models presented in Table 1: short known: accuracy = 0.56, precision = 0.56, recall = 0.52, f1 = 0.54 short unknown: accuracy = 0.58,precision = 0.57, recall = 0.62, f1 = 0.59 long unknown: accuracy = 0.64, precision = 0.59, recall = 0.55, f1 = 0.57. The models obtained from our research have a much higher ability to predict message credibility evaluations, which seems to be a specific brain process that cannot be explained as general decision making. This means that Hypothesis 6 is not supported.

We had designed the experiment of message credibility evaluation with consideration for internal validity. The task of Japanese language (Kanji signs) translation was chosen in order to exclude confounding variables, such as participant prior knowledge, experience or opinions on the subject of the evaluated message. We were also able to verify internal validity by evaluating the impact of one of the main independent variables (length of the note) on the participant's decisions (as stated by hypothesis 1). The experiment also had low attrition rate, as over 90% of participants completed the experiment.

The comparison of the models that predicted decisions in the cases with full knowledge (TT and FF) to the models that predicted cases with no knowledge, but under the influence of message design, shows great differences in the Brodmann areas selected by the machine learning algorithm as most significant for the prediction. This means that our results would allow to determine whether a person that evaluates message credibility bases this evaluation on what he knows (or believes that he knows). Note that in our experiment, participants accepted our translation of the three Kanji signs used in the full knowledge cases without verifying them).

In order to evaluate the external validity of our experiment, we can compare the results to results of a pilot experiment (Kawiak et al., 2020). The pilot experiment involved a different and smaller set of participants (57). It had a similar setting, but the long note and the short note were presented on a single screen. Hypotheses in the pilot experiment were similar to the ones described in this article, and their verification results are the same.

The Brodmann Areas used in the best classifiers for both experiments were different, because only one large time interval was taken into account in the pilot study, while in the main experiment we tested different smaller time intervals.

In the pilot study, short messages and long messages were displayed on the screen at the same time, making it impossible to determine whether the participant's response was connected to a positive message credibility evaluation of the first note or a negative evaluation of the second note (and vice versa). We have redesigned the main experiment to overcome this shortcoming by displaying short and long messages separately. This difference makes it difficult to directly compare models and results obtained in the pilot study and the main experiment. The results reported here regarding differences in brain activity during message credibility evaluation with and without prior knowledge are also missing in the pilot study (Kawiak et al., 2020).

Nevertheless, in the pilot study message credibility evaluation models used areas related to language processing and word comprehension such as BA38, BA39, BA40. In both experiments, there were classifiers that used areas like BA39—language reception and understanding and BA46—decision making under uncertainty.

Overall, we consider that the preliminary results in the pilot experiment confirmed the external validity of our experiment, because the results of hypothesis verification based on both experiments were the same.

Our experiment had several limitations. First, only right-handed, young men who were university students of a technical subject were included in our sample.

Second, our experiment controlled and limited the factors that could influence credibility evaluation. Only message credibility was available to experiment participants, who did not know the source of the message. While this setting resembled a situation in which a Web user evaluates credibility of content by an unknown author, the experimental setting was still very limiting. Other factors, such as the message look, persuasiveness, or emotional content, could influence message credibility. A limitation of the new method for selecting independent variables was the use of the same time interval for all Brodmann Areas for the sMEC calculations. The next step should be to search for different time intervals for different Brodmann Areas in a single model.

## 5. Conclusion and Future Work

Our results indicate that by using source localization algorithm (sLORETA) and an easy-to-interpret logistic regression algorithm, we can demonstrate and make use of the difference in brain activity during the decision making process to classify message credibility evaluations. This is an important first step toward a deeper understanding of human credibility evaluations. For instance, consider the credibility evaluation of debunking information designed to counteract fake news. The findings from this study can be used to guide the design of future experiments with a panel of judges who would evaluate the credibility of fake news or debunking information. Our results allow to determine, by observing the brain activity of such a judge, whether he made a credibility evaluation based on his knowledge (or what he believes to know), or not.

The next step is to investigate other cognitive biases that can affect message credibility evaluations, and to learn how to detect them using brain activity measurements. Our results show that this is a promising research direction.

In future work, we will also study the activity of parts of the brain in different time intervals and different frequency bands for each part of the brain, and to build even better classifiers of message credibility evaluation using more advanced models.

The study by Douglas et al. (2013) proposed models using various spectral bands of the collected signal, characterized by good accuracy. These models were not based on source location but on wavelets of EEG signals. In further work, we can also use different bands to increase the number of explanatory variables in the model.

The results of machine learning algorithms other than logistic regression may turn out to be better than the results of models presented in this article, at the expense of reduced interpretability. This is another direction of our future research.

## Data Availability Statement

The raw data supporting the conclusions of this manuscript will be made available by the authors, without undue reservation, to any qualified researcher.

## Ethics Statement

The studies involving human participants were reviewed and approved by the Maria Curie-Sklodowska University's Bioethical Commission (MCSU Bioethical Commission permission 13.06.2019). The patients/participants provided their written informed consent to participate in this study.

## Author Contributions

All authors listed have made a substantial, direct and intellectual contribution to the work, and approved it for publication.

## Conflict of Interest

The authors declare that the research was conducted in the absence of any commercial or financial relationships that could be construed as a potential conflict of interest.

## Publisher's Note

All claims expressed in this article are solely those of the authors and do not necessarily represent those of their affiliated organizations, or those of the publisher, the editors and the reviewers. Any product that may be evaluated in this article, or claim that may be made by its manufacturer, is not guaranteed or endorsed by the publisher.

## Footnotes

^1^All experiment participants had no knowledge of Japanese. Japanese language is not popular in Poland, it is not taught neither in schools nor at our University. Before the participation, students filled up the questionnaire in which they were asked to tick the appropriate level of knowledge of foreign languages as well as their age and others.

^2^Electrical Geodesic Systems, Inc., 500 East 4th Ave. Suite 200, Eugene, OR 97401, USA.

^3^The electrodes are described in EGI 256-channel cap specification as best for cognitive ERP observations, covering the scalp regularly, and numbered as follows: E98, E99, E100, E101, E108, E109, E110, E116, E117, E118, E119, E124, E125, E126, E127, E128, E129, E137, E138, E139, E140, E141, E149, E150, E151, E152. Those electrodes are automatically chosen for observing P-300 ERP signal by NetStation software.
